# Mechanical Properties of Nano-Crystalline Glass-Carbomer Cements Used in Dentistry

**DOI:** 10.3390/ma17051186

**Published:** 2024-03-04

**Authors:** Małgorzata Karolus, Adrian Barylski, Magdalena Fryc, Damian Strzelec

**Affiliations:** Institute of Materials Engineering, Faculty of Science and Technology, University of Silesia in Katowice, 75 Pułku Piechoty 1A, 41-500 Chorzów, Poland; adrian.barylski@us.edu.pl (A.B.);

**Keywords:** GCP glass fill, glass-carbomer cements, glass-polyalkene cements, tribology, Vickers hardness, SEM

## Abstract

The main aim of this study was to assess the impact of the environment on the mechanical and tribological properties of glass-carbomer cements used in dentistry. The properties of the Glass Cements Polyalkene (GCP) Glass Fill material, belonging to glass-polyalkene cements, were tested after placing it in various environments: air, distilled water, artificial saliva simulating a neutral environment (pH = 7), and simulating inflammation (pH = 4). The research material included four samples and a two-year reference material. The analysis of volumetric consumption and the assessment of the impact of solubility on the stability of glass-carbomer cements were carried out using tribological measurements and Vickers hardness measurements. In addition, microstructural characterization of the materials was performed using scanning electron microscopy (SEM). It was observed that the lowest wear (0.04%), the most stable microstructure, and the lowest average hardness (21.52 HV 0.1) were exhibited by the material stored in artificial saliva simulating a neutral environment (pH = 7). The least stable microstructure and statistically the highest hardness (77.3 HV 0.1) was observed in the test sample, which was stored in air for two years and then in distilled water. The highest consumption (0.11%) was recorded in the case of cement placed in artificial saliva simulating inflammation (pH = 4). The results obtained in this study indicate specific trends in the influence of the environment in which the tested cement is located, such as air, distilled water, air/distilled water, artificial saliva simulating a neutral environment, and simulating inflammation, on its structure, hardness, and wear.

## 1. Introduction

Modern dentistry focuses on the development of minimally invasive treatment and prevention. The presented results were based on the use of innovative methods of preparing cavities that preserved hard tissues and reconstruction techniques using materials adhesive to the tooth structure. Such materials include glass-ionomer cements, glass-carbomer cements, composites, compomers, ormocers, and giomers, which are also adequate in terms of aesthetics and strength. In cases of primary caries treatment of permanent teeth, it is recommended to use them first, remembering to maintain appropriate indications for their use [1].

Choosing a specific restorative material involves individualizing the treatment and should be based on knowledge of the composition, properties, and characteristics of the material. Conditions such as the preferences of the treated person, the ability to control moisture during the procedure, the degree of advancement of the caries lesion, and the level of oral hygiene are also taken into account. The dentist has at his disposal preventive and restorative adhesive materials in tooth color, which include the following: resin-modified glass-ionomer cements, conventional glass-ionomer cements, glass-carbomer cements, composite materials, composite materials modified with polyacid (compomers), ormocers, and giomers. In the near future, further development of minimally invasive techniques is expected, in particular regarding the search for improvements in the field of restorative materials, primarily in terms of their mechanical properties, such as abrasion resistance, strength, and biocompatibility [1]. The group of polymeric materials, the newest fillings used to rebuild hard tooth tissues, includes, among others, resin-based composites, compomers, giomers, ormocers, glass-ionomer cements, including their resin varieties, and glass-carbomer cements [2]. In the literature, you can find observations made on other various materials using methods that constitute the basis for the analyses conducted in this work. Thus, in 2017, Doğan et al. found high wear resistance of the analyzed material, which is also reflected in the tribological test results presented in the paper [3]. In 2016, Lopes et al. reported an average GCP microhardness value of 69.05 HV 0.1, which is similar to the average measured values of Vickers hardness collected above [4]. In 2015, Arslanoglu et al. observed defects and cracks on the surface of glass-carbomer cement in photomicrographs and assessed the smoothness as very good, confirming the observations made based on the analysis of SEM images [5].

A wide selection of various types of dental materials and their mechanical properties are described in the works [6,7,8]. The tribological properties, hardness, and microstructure of the materials were compared there.

Glass-carbomer cements were relatively recently introduced into use (2005) by the Dutch company GCP. According to commercial terminology, carbomer is treated as a water-absorbing homopolymer of poly(acrylic acid)—(CH_2_-CH(COOH))_n_-(24). They are classified as glass-polyalkene cements and are characterized by higher bioactivity, acid resistance, and remineralization power than standard GIC [9]. They are 100% biocompatible and safe for the dentist, the patient, and the environment throughout the entire cycle of production, processing, and loss of ions during the wear process. They are used as substitutes for glass-ionomer cements and other composites used in restorative dentistry. They are used for the renovation of primary teeth, reconstruction of bridges and crowns, and permanent reconstruction of cavities using heat [10]. One of the promising methods to improve the physical and mechanical properties of GIC composites is the introduction of various types of admixtures. In work [11], the authors enriched the GIC cement preparation with micrometric gum arabic (GA) powder of various concentrations using two commercially available GIC cement materials (Medicem and Ketac Cem Radiopaque). It was found that the inclusion of oxidized GA powder in the GIC formulation helps to improve mechanical properties with a slight increase in water solubility and sorption parameters.

In terms of structure, these materials do not differ from glass-ionomer cement, but they differ in additives in their composition, such as hydroxyapatite or fluoroapatite, which improves the remineralization process. Their nanocrystals act as a second bioactive filler and are added in an amount of up to 20% by weight, and up to 50% by weight in the sealant of grooves and holes, as remineralization nuclei with the intention of forming enamel-like material at the tooth border [9]. They are sometimes carbomined with nanoparticles containing glass-ionomer cements reinforced with a specially designed filler and FAp particles. They do not contain metals, solvents, and resins and are monomer-free [10].

Due to only preliminary clinical tests, their durability in the human oral cavity has not yet been determined. They are less soluble and have higher compressive strength (260 MPa) and bending strength (122 MPa) as well as wear and shear resistance (13.7 MPa) compared to glass-ionomer cements [9]. The stresses occurring in them are compressive, which has a positive effect on the fatigue resistance of the material when exposed to variable and periodic forces, such as chewing, and also prevents the initiation and propagation of cracks in the material, which significantly extends its service life. The lower roughness of their surface is associated with a lower value of nanohardness and reduced Young’s modulus. They have a slight hardness of 0.57 GPa. Due to their fragility, the average value of the reduced Young’s modulus is burdened with a higher measurement error [10]. The consequence of incorporating fluorapatite nanocrystals into their structure may be increased instability [9].

Research conducted in laboratory conditions on composite dental materials used in regenerative techniques is widely described in the literature [12,13,14]. An example of such research may be the analysis of mechanical fracture resistance and correlations with the wear behavior of hydroxyapatite and silica/hydroxyapatite filled bis-GMA/TEGDMA micro/hybrid dental composites [14]. The authors found that wear, ductility, and hardness values have a correlation with each other, which is due to the direct relationship between the brittleness parameter and specific wear rates. The worn surface morphology showed strong traction marks for the highly filled composites (H50 and SH50). It was indicated that it is important to design fracture- and wear-resistant hybrid and microfilled dental composites by controlling the mechanical properties.

Most published results refer to the characterization of the morphology and mechanical properties of dental composites under theoretical conditions [15,16], or they refer to the simulation of phenomena present in the materials, such as stresses leading to material wear. Therefore, it is important to refer and verify the research results to real conditions. Due to the lack of in-depth knowledge regarding the actual consumption of polyalkene materials (GCP) from the glass cement industry in the body’s environment, this study attempts to investigate selected material properties, i.e., microstructural characteristics and tribological properties of materials stored in conditions similar to real ones, i.e., in the environment neutral, in artificial saliva simulating a neutral state, and in inflammation.

An innovative approach to research is the analysis of material exposed to long-term environmental effects, including solutions simulating artificial saliva in a neutral and inflammatory state. It is also interesting to compare the results with a sample stored in air. The ability to perform measurements on two-year-old samples provides a significant contribution to the knowledge regarding the stability and durability of dental materials. Such a comparison gives an idea of the influence of the type of environment in which the material is kept. By comparing the level of consumption of materials stored in different environments and over different periods of time, they allow conscious selection of appropriate materials for treatment in neutral conditions and appropriate for the treatment of inflammatory conditions. The main goal of the article was to compare the strength results of dental materials with conditions resembling the real environment of the mouth and bite work. An additional advantage of the job was the opportunity to examine material covered by the internship for two years. The research is of a preliminary nature, introducing the issue, which will be analyzed in more detail in the future.

## 2. Materials and Methods

The main aim of this study was to assess the impact of the environment on the mechanical properties of glass-carbomer cements used in dentistry. The work has included the preparation of research material, i.e., GCP containing HAp and FAp nanoparticles, and preparation of the environments in which the samples were stored: artificial saliva with different pH, simulating a neutral environment (pH = 7), and simulating inflammation (pH = 4) and distilled water as well as performing a tribological test, Vickers hardness tests and microstructure analysis using SEM.

Test samples were prepared from the GCP Glass Fill material by GCP Dental according to the same procedure in accordance with the manufacturer’s recommendations [17]. The material enclosed in the capsule goes to a high-frequency mixer, where it is mixed for 10–15 s, after which the material is activated in the capsule gun within 15 s. The procedure was performed under normal room conditions. Light cure samples on each side of a high-energy lamp for a total of approximately one minute. Each of the received samples was placed in a separate container in an appropriate environment until testing began. Finally, all of them were placed in an incubator that maintains the temperature of the human body (approx. 37 °C). All obtained samples were formed into tablets with a diameter of φ = 10 mm and a height of d = 5 mm. All tests were performed for 4 samples of each material.

The environments in which the samples were stored, i.e., artificial saliva with different pH, simulating a neutral environment (pH = 7), and simulating inflammation (pH = 4), were prepared in accordance with the PN-EN ISO 10271 standard (Table 1) [18].

**Figure 1:**
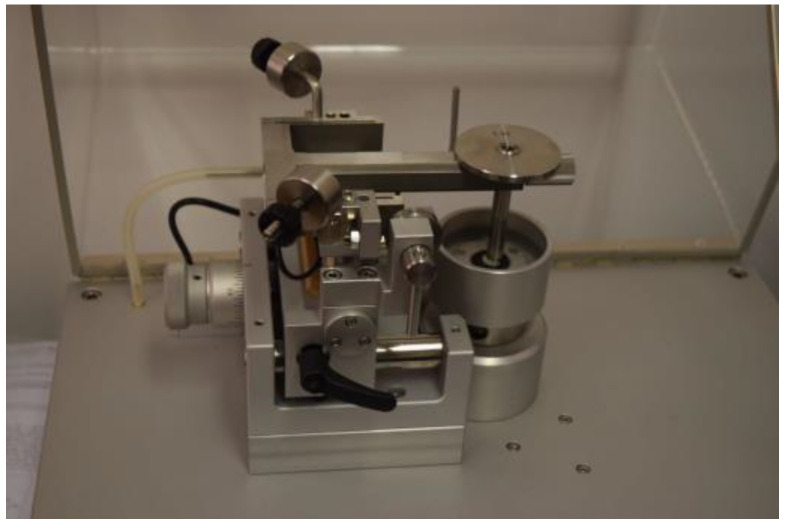
Anton Paar TRN-type tribometer and exemplary tribological test parameters.

In order to consider aspects related to the mechanical strength of cements, measurements of wear, hardness, and morphology were performed. The TRN-type tribometer device with the InstrumX software 7.3.17 (Anton Paar, Corcelles-Cormondrèche, Switzerland) allowed the determination of material wear (Figure 1). A 6 mm Al_2_O_3_ ceramic ball was used as a counter sample. The following cycle was planned in the software intended for the device: continuous rotation around a radius of 1.5 mm, with a linear speed of 1.25 cm/s, with a load of 2 N and a frequency of 50 Hz, for a distance of 4000 laps in the environment rich in air, with a temperature of 21 °C and humidity of 40% (Table 2).

The average area of the wear track, P, was determined using a Surftest SJ-500 profilometer (Mitutoyo, Tokyo, Japan). Surface area measurements were averaged for four 2D profiles acquired every 90°. Volumetric wear, V_W_, was determined from the following Formula (1):

(1) $$V_{W}=\frac{V}{F_{N}\cdot s}\left[\frac{{\mathrm{mm}}^{3}}{\mathrm{Nm}}\right]$$

where *F_N_* is the load applied [N], *s* is friction distance [m], *V* is the volume of the wear track calculated from the formula *V* = P·2πr [mm^3^], *P* is the average area of the wear track [mm^2^], and *r* is the friction distance radius [mm].

Using a Wolpert Wilson Instruments microhardness tester model 401 MVD type (Wolpert Wilson, Worcester, MA, USA), the Vickers hardness was determined in the range of the microhardness scale (designation HV 0.1), where a load of 0.98 N was used. The impact of the indenter lasted as short as possible (10 s) (Table 3). In total, 5 measurements were made for each sample. To examine the microstructure, a JSM-6480 scanning electron microscope from JEOL (Tokyo, Japan) was used. An analysis was made of both the area subjected to the tribological test and the area free from signs of wear. The resulting images were magnified 25 or 100 times with a resolution of 1 mm and 100 μm (Table 4). In each case, the samples were tested separately, one after the other, under the same laboratory conditions.

The morphology and structure characterization of the obtained samples were examined using the JSM-6480 firmy JEOL scanning electron microscopes (SEM). For each sample, 5 measurements were made.

## 3. Results

### 3.1. Tribology

The result of the tribological test is data on the consumption of the GCP Glass Fill dental material. Table 5 shows the average area of wear tracks (Table 5). The highest value was found in the case of a sample stored in artificial saliva simulating inflammation (pH = 4), 0.060 mm^2^, while the lowest value was found in the test sample, 0.033 mm^2^. The remaining measured values were 0.052 mm^2^ for the material placed in artificial saliva simulating a neutral environment (pH = 7) and 0.038 mm^2^ for cement stored in distilled water. The average areas of wear tracks were derived from measurements of 4 samples (Table 5).

Analyzing the volumetric wear, by far the highest value is found in the case of the test sample, 0.0165 mm^3^/Nm, while the lowest in the case of GCP stored in artificial saliva simulating a neutral environment (pH = 7), 0.0065 mm^3^/Nm, and distilled water, 0.0048 mm^3^/Nm (3.4 times reduction in wear compared to test sample). A slightly higher value of 0.0074 mm^3^/Nm was measured for the material placed in artificial saliva simulating inflammation (pH = 4) (Table 6).

### 3.2. Friction

The highest value of the friction coefficient was recorded in a two-year-old sample (reference sample), 1.376, while the lowest value was recorded in a sample stored in artificial saliva simulating inflammation (pH = 4), 0.423. The remaining measured values are 0.98 for cement in distilled water and 1.097 for the material placed in artificial saliva simulating a neutral environment (pH = 7) (Table 7 and Table 8).

The test sample is characterized by the highest average friction coefficient of 0.855, while the smallest friction coefficient determined for the sample stored in artificial saliva simulating inflammation (pH = 4) is 0.352. In the case of GCP placed in distilled water or artificial saliva simulating a neutral environment (pH = 7), the values obtained were 0.813 and 0.648, respectively. Due to the variability of this parameter, the measurement uncertainty was also determined, for which the maximum fluctuations were found for two-year-old material (±0.11), and the minimum for the material stored in distilled water (±0.03). The remaining values of deviations were recorded for cements found in artificial saliva simulating inflammation (±0.04) and simulating a neutral environment (±0.08) (Table 7).

Based on the changes in the friction coefficient during the test, the greatest variability of this parameter is recorded in the test sample, while the smallest is in the case of GCP stored in distilled water. Variations in the stability of the friction coefficient are also observed in materials placed in artificial saliva simulating inflammation (smaller) and simulating a neutral environment (larger). The curves for both the test cement and the cement stored in artificial saliva simulating a neutral environment (pH = 7) are similar, reaching maximum values already at the beginning of the cycle, to reach a relative equilibrium at a lower level after a few minutes. The course is slightly different for material stored in artificial saliva simulating inflammation (pH = 4), where the values initially increase and then stabilize. For the sample stored in distilled water, the distribution of the results obtained is stable. The recorded friction coefficients vary in the following ranges:

- 0.45–0.95 for GCP in distilled water;
- 0.55–1.10 for GCP in artificial saliva simulating a neutral environment;
- 0.20–0.40 for GCP in artificial saliva simulating inflammation (least spread);
- 0.45–1.40 for two-year GCP (largest spread).

### 3.3. Vickers Hardness Measurements

By carrying out Vickers hardness measurements, the material’s resistance to deformation was determined. Table 9 shows the average hardness values for each sample. GCP for testing purposes achieved the best result, 77.3 HV 0.1, while GCP stored in artificial saliva simulating a neutral environment (pH = 7) had the worst result, 21.52 HV 0.1. In the case of cement in air, the hardness was 71.44 HV 0.1, in distilled water, 25.7 HV 0.1, and in artificial saliva simulating inflammation (pH = 4), 24 HV 0.1.

To determine the measurement uncertainty (standard deviations), the measurements were performed five times. The maximum fluctuations were recorded in the sample placed in artificial saliva simulating inflammation (±3.19), and the minimum in the case of cement stored in distilled water (±1). The remaining variability measurement results were attributed to two-year-old material (±2.81), kept in the air (±1.03), and artificial saliva simulating a neutral environment (±1.11) (Table 9).

### 3.4. SEM Analysis

**Figure 2:**
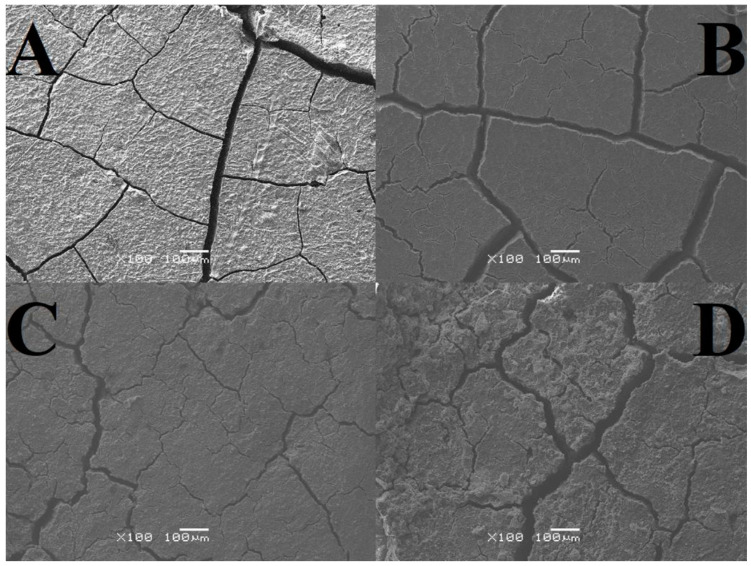
Images of microstructures showing the area not subjected to tribological tests; (**A**) sample stored in distilled water, (**B**) sample stored in artificial saliva simulating a neutral environment (pH = 7), (**C**) sample stored in artificial saliva simulating inflammation (pH = 4), (**D**) sample stored in air/distilled water for 2 years (SEM images were taken at 100× magnification).

**Figure 3:**
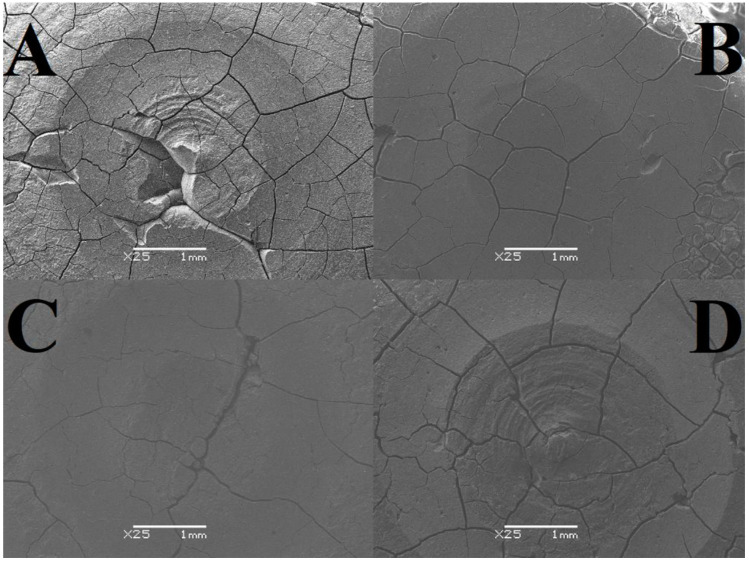
Images of microstructures showing the area of the entire abrasion trace after tribological tests; (**A**) sample stored in distilled water, (**B**) sample stored in artificial saliva simulating a neutral environment (pH = 7), (**C**) sample stored in artificial saliva simulating inflammation (pH = 4), (**D**) sample stored in air/distilled water for 2 years (SEM images were taken at 25× magnification).

**Figure 4:**
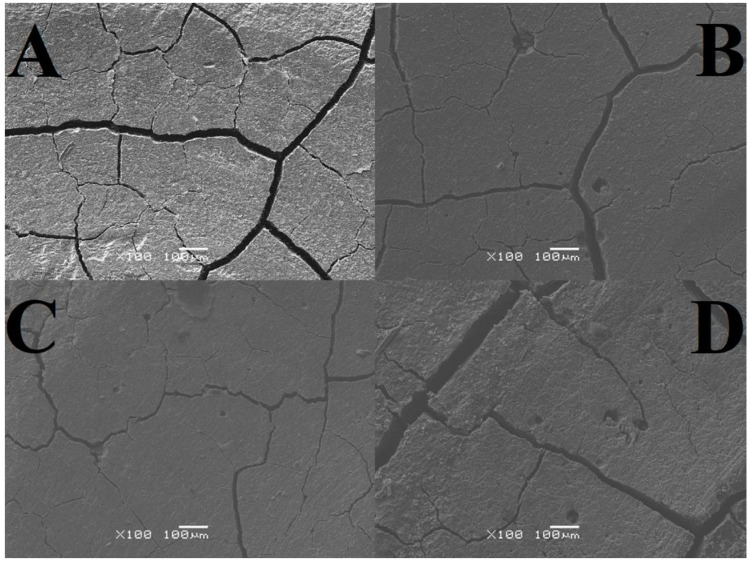
Microstructure images showing a close-up area of the wear mark; (**A**) sample stored in distilled water, (**B**) sample stored in artificial saliva simulating a neutral environment (pH = 7), (**C**) sample stored in artificial saliva simulating inflammation (pH = 4), (**D**) sample stored in air/distilled water for 2 years (SEM images were taken at 100× magnification).

As a result of the SEM analysis, images of GCP microstructures were obtained, both in the area subjected to tribological testing (including its close-up) and without signs of wear (Figure 2, Figure 3 and Figure 4). The tables present average crack widths measured using the ImageJ program (https://imagej.net/ij/, accessed on 29 February 2024, Table 10).

Based on the results obtained, it can be concluded that all tested samples have pores and cracks in various numbers and sizes, which proves the high heterogeneity of each of the analyzed surfaces. The highest cohesion was observed for cement stored in air, while the lowest was observed for the test material. We can distinguish cement stored in artificial saliva simulating a neutral environment (pH = 7), where gaps and unevenness occur to a minimum, unlike the test material (two-year-old). As for significant differences, changes occur in the two-year-old sample in the form of intensively forming sediment. Analyzing the abrasion traces, the most visible wear is observed in the case of the test material and the one stored in distilled water, while for both cements in artificial saliva, it is barely noticeable (Figure 2, Figure 3 and Figure 4).

The highest crack value was recorded for the test sample, 0.007 mm, while the smallest was found for GCP stored in artificial saliva simulating a neutral environment (pH = 7), 0.003 mm. Very similar results, amounting to 0.004 mm and 0.005 mm, respectively, were achieved by material placed in artificial saliva simulating inflammation (pH = 4) and cement placed in distilled water. The sample stored in artificial saliva simulating inflammation (pH = 4) had the lowest variability (±0.0006), while the remaining three GCPs had identical fluctuations (±0.0007) (Table 10).

## 4. Discussion

The results obtained in this study provide insight into the real consumption of GCP composite dental cement in conditions simulating both a neutral oral environment (artificial saliva) and an environment simulating inflammation (artificial saliva with pH = 4). In the above conditions, selected material properties were examined, i.e., microstructure and tribological properties. The analysis presented in this work showed a comparison of wear in individual environments based on average areas of abrasion traces, mass losses, volumetric wear, maximum and average friction coefficients, changes in the friction coefficient during the set cycle, and wear depth profiles. The results of tribological tests presented in this paper made it possible to determine how much loss the analyzed material suffers when the basic forces acting on it, such as chewing, occur, which allows for the assessment of the filling efficiency. Completing the test after 4000 laps gives the opportunity to evaluate materials that are 4 years older than they actually are (it is assumed that 1000 laps correspond to aging by 1 year).

Analyzing the average areas of the abrasion trace, it can be noticed that these values are not large in relation to the total area. You can see the dependence of the obtained values on the environment in which the material was stored. The lowest values of the worn surface, i.e., 0.033 mm^2^ and 0.038 mm^2^, were obtained for two-year-old material stored in air/distilled water and in distilled water, while the highest values, i.e., 0.052 mm^2^ and 0.06 mm^2^, characterize cements placed in conditions intended to simulate the natural environment, i.e., artificial saliva simulating a neutral environment (pH = 7) and simulating inflammation (pH = 4). There is almost a two-fold difference between the sizes of the largest and smallest areas, which clearly indicates the strong influence of real conditions on material consumption.

The changes in the masses of all GCPs are small; however, it can be observed that the largest mass loss occurred for the material stored in artificial saliva simulating inflammation (pH = 4), 0.11%, while the smallest reduction occurred for the cement located in the same environment simulating a neutral environment (pH = 7), 0.04%. The value determining the reduction in the mass of the sample placed in distilled water, i.e., treated as a reference point, is 0.07%, which represents the middle value of losses.

Volumetric consumption is also characterized by very small values, but unlike previous measurements, two samples—stored in artificial saliva simulating inflammation (pH = 4) and distilled water—were characterized by the lowest volume loss—0.005 mm^3^/Nm. GCP contained in artificial saliva simulating a neutral environment (pH = 7) showed an imperceptibly larger volume loss—0.006 mm^3^/Nm. In turn, the test material most affected by wear suffered three times more than the others—0.017 mm^3^/Nm.

Vickers hardness measurement is used to assess the strength of a material subjected to specific pressures during its use. The parameters were selected based on the use of glass-carbomer cements in dentistry described in the literature [7,8,12]. Due to the heterogeneity of the material, the results obtained differ from typical values; however, the obtained results provide valuable information regarding the nature of changes occurring in materials stored in various environments. The test sample is characterized by the highest hardness, 77.3 HV 0.1, similar to the GCP found in air, 71.44 HV 0.1. However, cement stored in artificial saliva simulating a neutral environment (pH = 7) has the lowest resistance, 21.52 HV 0.1, similar to the material placed in the same environment simulating inflammation (pH = 4), 24 HV 0.1, and the sample in distilled water, 25.7 HV 0.1. A clear weakening of GCPs immersed in liquids is noticeable. However, considering the fact that in the case of material stored in artificial saliva simulating inflammation (pH = 4), the greatest weight loss is observed at the same time, it can be assumed that these effects are related to the processes of releasing the composite material for the treatment and regeneration of diseased tissue.

The analysis of SEM images shows that the surface of the cement in the air is almost free of cracks and pores, and those noted are barely noticeable. There are numerous irregularities in the form of thickenings and recesses, but due to its compact structure, it can be considered the best. The outer layer of GCP placed in artificial saliva simulating a neutral environment (pH = 7) looks different, where there are minor plane disruptions, still few pores, but more large cracks with a width of 0.003 mm. In the case of the sample surface stored in the same environment simulating inflammation (pH = 4), slightly wider and more numerous gaps with a width of 0.004 mm, as well as more irregularities, can be seen. Samples of the material, taken as a reference point, located in distilled water, especially the test one, are characterized by increasingly larger cracks with widths of 0.005 mm and 0.007 mm, respectively, and more frequently occurring pores of increased size or structure disruptions. Additional confirmation of this order are the microhardness results, because the higher its value, the greater the roughness. To sum up, it can be said that the artificial saliva environment has a positive effect on the condition of the tested GCP surfaces. Ultimately, all surfaces can be considered good enough to perform their functions. The main differences include the sediment formed on two-year-old cement and the “shiny” image of the material placed in distilled water. The first phenomenon may be related to the formation of apatite fractions, while the second one may be related to the formation of the matrix.

The results indicating the possibility of apatite formation in glass-carbomer cement and the fact that the material contains its fraction even after 10 months bode well for the technological development of biomaterials for direct restorations. However, as only preliminary clinical tests have been carried out, their durability in the human oral cavity has not yet been determined. Generally, they are less soluble and have higher compressive strength (260 MPa) and flexural strength (122 MPa) as well as wear and shear resistance (13.7 MPa) compared to glass ionomer cements. The stresses occurring in them are compressive, which has a positive effect on the fatigue resistance of the material when exposed to variable and periodic forces, such as chewing, and also prevents the initiation and propagation of cracks in the material, which significantly extends its service life. Therefore, in the future, it is recommended to carry out tests such as determining the residual stresses or compressive strength of the materials or repeating similar observations while modifying the storage time of the material and using the silicone coating recommended by the manufacturer.

Various types of tests on the properties of dental materials are described in the literature. However, most of them refer to typical laboratory tests. In this article, an effort was made to ensure that test materials were tested in a simulation environment, with actual wear conditions of dental materials of a specific type. An innovative approach to research is the analysis of material exposed to long-term environmental effects, including solutions simulating artificial saliva in a neutral and inflammatory state. It is also interesting to compare the results with a sample stored in air. The ability to perform measurements on two-year-old samples provides a significant contribution to the knowledge regarding the stability and durability of dental materials. Such a comparison gives an idea of the influence of the type of environment in which the material is kept. By comparing the level of consumption of materials stored in different environments and over different periods of time, they allow conscious selection of appropriate materials for treatment in neutral conditions and appropriate for the treatment of inflammatory conditions. Nevertheless, the results of this article should be treated as preliminary research leading to further tests, which will involve a detailed comparative analysis of materials of different ages, simulating different storage environments.

**Figure 5:**
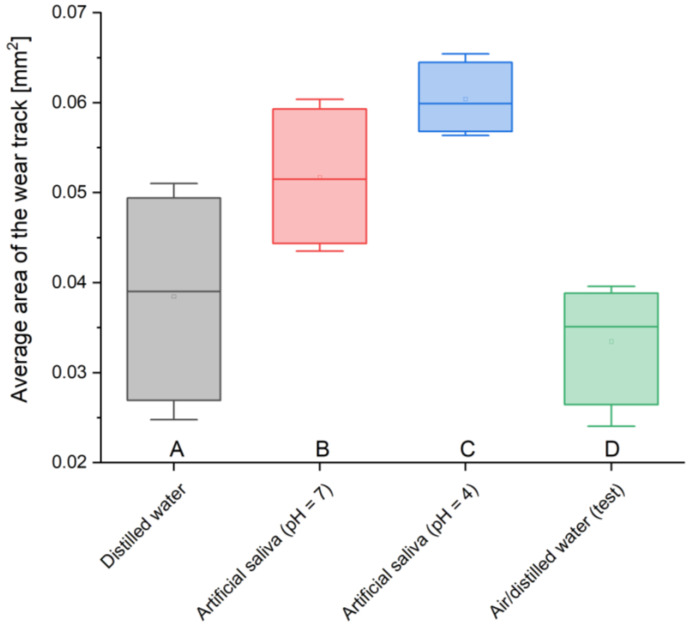
Average area of the wear track box plot; (**A**) sample stored in distilled water, (**B**) sample stored in artificial saliva simulating a neutral environment (pH = 7), (**C**) sample stored in artificial saliva simulating inflammation (pH = 4), (**D**) sample stored in air/distilled water for 2 years.

**Figure 6:**
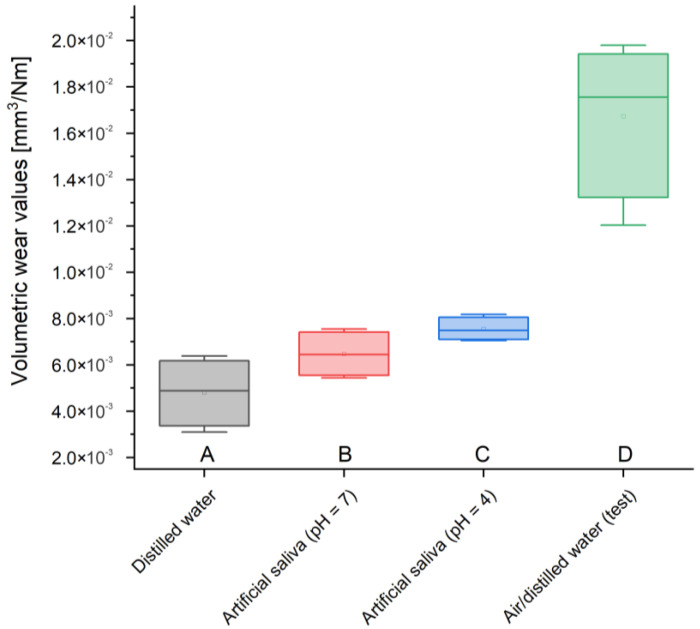
Volumetric wear values box plot; (**A**) sample stored in distilled water, (**B**) sample stored in artificial saliva simulating a neutral environment (pH = 7), (**C**) sample stored in artificial saliva simulating inflammation (pH = 4), (**D**) sample stored in air/distilled water for 2 years.

**Figure 7:**
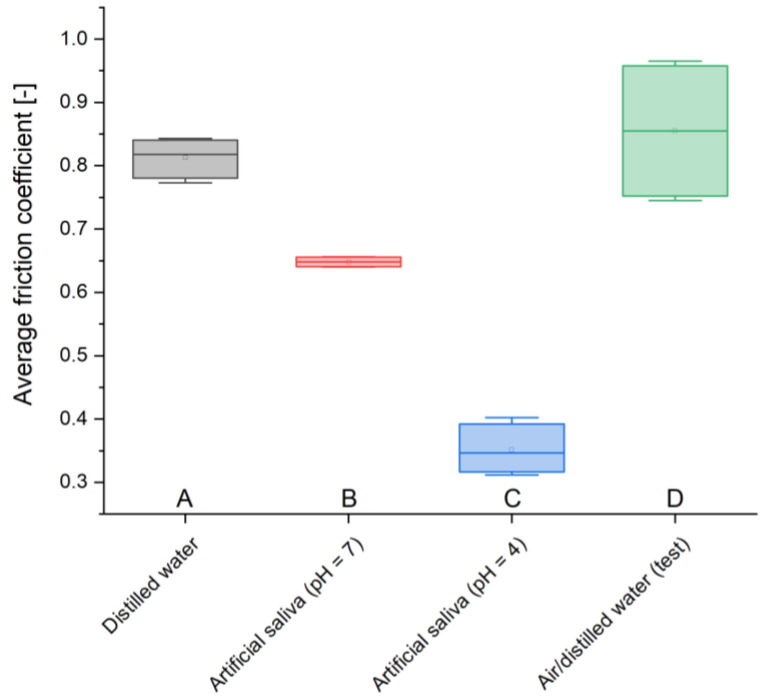
Average friction coefficient box plot; (**A**) sample stored in distilled water, (**B**) sample stored in artificial saliva simulating a neutral environment (pH = 7), (**C**) sample stored in artificial saliva simulating inflammation (pH = 4), (**D**) sample stored in air/distilled water for 2 years.

**Figure 8:**
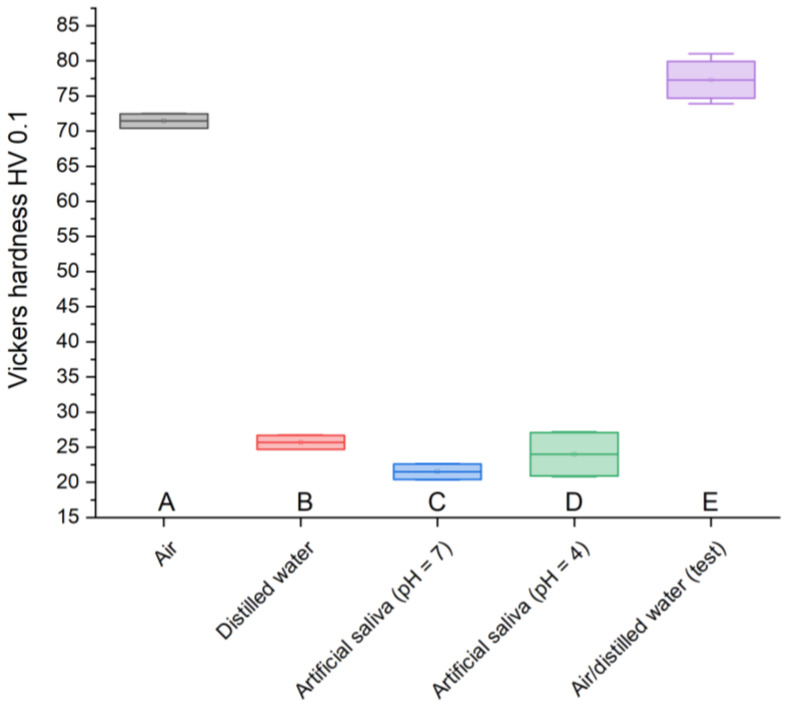
Vickers hardness box plot; (**A**) sample stored in air, (**B**) sample stored in distilled water, (**C**) sample stored in artificial saliva simulating a neutral environment (pH = 7), (**D**) sample stored in artificial saliva simulating inflammation (pH = 4), (**E**) sample stored in air/distilled water for 2 years.

To summarize the results obtained in this work, it is necessary to refer to the statistical analysis of the results and the number of measurement samples carried out. The results of many engineering tests and ISO standards presented in the literature show that even a small number of samples is considered sufficient and provides reliable results. An example of this could be tribological tests, also carried out in this work, where a sufficient number of repetitions is four. However, due to the small number of samples, statistical analysis was carried out using the ANOVA test. The results obtained for individual analyses are presented in the following charts (Figure 5, Figure 6, Figure 7 and Figure 8).

## 5. Conclusions

Each of the environments, air, distilled water, air/distilled water, and artificial saliva simulating a neutral environment and simulating inflammation, has a different impact on specific properties of the sample, and, therefore, it is impossible to clearly determine which environment has the most beneficial effect and which ones are the most negative. However, the obtained results show specific trends in the impact of the environment in which the tested cement is located on its structure, hardness, and wear.

The influence of the environment on the mechanical properties of GCP Glass Fill glass-carbomer cements used in dentistry can be presented in the form of the following conclusions:

- the highest signs of wear (0.11%) were found in the case of storing GCP in artificial saliva simulating inflammation (pH = 4) and the smallest (0.04%) in the material also stored in artificial saliva but simulating a neutral environment (pH = 7);
- the highest microhardness (77.3 HV 0.1) was found for the test sample, i.e., stored in air and distilled water, while the lowest (21.52 HV 0.1) was found for cement placed in artificial saliva simulating a neutral environment (pH = 7);
- the best morphological surface (little differentiation, barely visible trace of abrasion, the narrowest cracks) was found in the case of GCP located in artificial saliva simulating a neutral environment (pH = 7), and the worst (large differentiation, visible trace of abrasion and sediment, the widest cracks) was a two-year sample stored in air and distilled water.

## Figures and Tables

**Table 1:** Chemical composition of artificial saliva based on the PN-EN ISO 10271 standard [18].

Compound	Quantity (g)
K_2_HPO_4_	0.20
Na_2_HPO_4_	0.26
KSCN	0.33
NaHCO_3_	1.50
NaCl	0.70
CO(NH_2_)_2_	0.13
KCl	1.20

**Table 2:** Parameters characterizing tribological testing.

Parameter	Value
Type, geometry, size of the counter-sample	Al_2_O_3_, ball, 6 mm
The nature of the movement	fixed, rotatable
Track radius	1.5 mm
Linear speed	1.25 cm/s
Load	2 N
Frequency	50 Hz
Distance to cover	4000 laps
Environmental conditions	air, temperature 21 °C, humidity 40%

**Table 3:** Parameters characterizing the measurement of Vickers hardness.

Parameter	Value
Scale range	HV 0.1
Load	0.98 N
Impact time	10 pp

**Table 4:** Parameters characterizing SEM analysis.

Parameter	Value
Accelerating voltage	≤20 keV
Magnification	25× or 100×
Resolution	1 mm or 100 μm

**Table 5:** Values of the average area of the wear track.

Sample Storage Environment	Number of Measurements	Mean Area of the Wear Trace	Standard Deviation	Signifiance Level	Confidence Interval
	n	P (mm^2^)	σ (mm^2^)	α	CI
Distilled water	4	0.038	0.012	0.05	0.012
Artificial saliva (pH = 7)	4	0.052	0.008	0.05	0.008
Artificial saliva (pH = 4)	4	0.060	0.004	0.05	0.004
Air/distilled water (test)	4	0.033	0.007	0.05	0.007

**Table 6:** Volumetric wear values.

Sample Storage Environment	Number of Measurements	Mean Value of Volumetric Wear	Standard Deviation	Signifiance Level	Confidence Interval
	n	Vw (mm^3^/Nm)	σ (mm^3^/Nm)	α	CI
Distilled water	4	0.0048	0.0015	0.05	0.0015
Artificial saliva (pH = 7)	4	0.0065	0.0011	0.05	0.0011
Artificial saliva (pH = 4)	4	0.0074	0.0004	0.05	0.0004
Air/distilled water (test)	4	0.0165	0.0036	0.05	0.0035

**Table 7:** Values of the average friction coefficient.

Sample Storage Environment	Number of Measurements	Average Value of Coefficient of Friction	Standard Deviation	Signifiance Level	Confidence Interval
	n	μ_mean_ (-)	σ (-)	α	CI
Distilled water	4	0.813	0.030	0.05	0.029
Artificial saliva (pH = 7)	4	0.648	0.008	0.05	0.008
Artificial saliva (pH = 4)	4	0.352	0.040	0.05	0.039
Air/distilled water (test)	4	0.855	0.110	0.05	0.108

**Table 8:** Values of the maximum friction coefficient.

Sample Storage Environment	Max. Coefficient of Friction
Distilled water	0.980
Artificial saliva (pH = 7)	1.097
Artificial saliva (pH = 4)	0.423
Air/distilled water (test)	1.376

**Table 9:** Average Vickers hardness values.

Sample Storage Environment	Number of Measurements	Average Value of Vickers Hardness	Standard Deviation	Signifiance Level	Confidence Interval
	n	(HV 0.1)	σ (HV 0.1)	α	CI
Air	5	71.44	1.03	0.05	0.90
Distilled water	5	25.70	1.00	0.05	0.88
Artificial saliva (pH = 7)	5	21.52	1.11	0.05	0.97
Artificial saliva (pH = 4)	5	24.00	3.19	0.05	2.80
Air/distilled water (test)	5	77.30	2.81	0.05	2.46

**Table 10:** Values of average crack width.

Sample Storage Environment	Number of Measurements	Average Value of Crack Width	Standard Deviation	Signifiance Level	Confidence Interval
	n	(mm)	σ (mm)	α	CI
Distilled water	5	0.005	0.0007	0.05	0.0006
Artificial saliva (pH = 7)	5	0.003	0.0007	0.05	0.0006
Artificial saliva (pH = 4)	5	0.004	0.0006	0.05	0.0005
Air/distilled water (test)	5	0.007	0.0007	0.05	0.0006

## Data Availability

Data are contained within the article.
